# The Underexploited Role of Non-Coding RNAs in Lysosomal Storage Diseases

**DOI:** 10.3389/fendo.2016.00133

**Published:** 2016-09-21

**Authors:** Matheus Trovão de Queiroz, Vanessa Gonçalves Pereira, Cinthia Castro do Nascimento, Vânia D’Almeida

**Affiliations:** ^1^Laboratório de Erros Inatos do Metabolismo, Department of Psychobiology, Universidade Federal de São Paulo, São Paulo, Brazil; ^2^Department of Pediatrics, Universidade Federal de São Paulo, São Paulo, Brazil

**Keywords:** non-coding RNA, lysosomal storage disease, lysosome, miRNA, siRNA

## Abstract

Non-coding RNAs (ncRNAs) are a functional class of RNA involved in the regulation of several cellular processes which may modulate disease onset, progression, and prognosis. Lysosomal storage diseases (LSD) are a group of rare disorders caused by mutations of genes encoding specific hydrolases or non-enzymatic proteins, characterized by a wide spectrum of manifestations. The alteration of ncRNA levels is well established in several human diseases such as cancer and auto-immune disorders; however, there is a lack of information focused on the role of ncRNA in rare diseases. Recent reports related to changes in ncRNA expression and its consequences on LSD physiopathology show us the importance to keep advancing in this field. This article will summarize recent findings and provide key points for further studies on LSD and ncRNA association.

## Lysosomal Storage Diseases

Lysosomal storage diseases (LSD) are a group of inherited diseases due to deficiency of lysosomal proteins such as hydrolases, membrane proteins, and lysosomal accessory proteins, which causes blockage in specific lysosomal catabolic pathways and storage of undegraded substrates. Lysosomal disturbance impairs relevant cellular functions, such as autophagy and vesicle trafficking, leading to accumulation of dysfunctional organelles and activation of secondary cascades which may affect several processes (1). To date, more than 50 LSD have been described, all of them inherited as autosomal recessive, with the exception of Danon disease, Fabry disease, and mucopolysaccharidosis type II, which are X-linked (2, 3).

All LSD are chronic, multisystemic, and progressive disorders, with a combined prevalence estimated of 1:8000 live births (2). Almost two-thirds of patients present neurological alterations, putting the group of LSD among the most common causes of neurodegeneration (4). Neuropathology in LSD usually develops from unique temporal and spatial changes, leading to early neurodegeneration and inflammation in specific regions, before global brain involvement (1). Visceral, ocular, hematological, and skeletal manifestations are also common [reviewed by Boustany (5)]. Signs and symptoms vary considerably depending on the disease, as well as age of onset and severity; some patients die before the first decade of life, whereas others may present normal lifespan. Phenotypic variability occurs not only between different LSD but also between patients with the same disease, even from the same family.

Allelic heterogeneity is a common feature of LSD; a high number of mutations have already been identified in the respective genes, hampering genotype–phenotype correlations. Although the primary biochemical and genetic defects are currently known for most LSD, this monogenic view is not enough to explain their physiopathology. The type and amount of undegraded (or partially degraded) substrates, along with the type and function of affected cells are important factors to determine cellular consequences of the specific enzyme deficiency. In addition, lysosomal dysfunction has an impact on several basic biological processes; recent studies have demonstrated the role of lysosomes in different cellular functions, such as vesicle trafficking, autophagy, nutrient sensing, cellular growth, and signaling, showing that they are not only catabolic organelles [reviewed by Parenti et al. (6)]. In fact, lysosomal alterations have been associated with pathogenic mechanisms in other neurodegenerative diseases such as Huntington’s, Parkinson’s, and Alzheimer’s, not only in LSD (7–9) but also due to the role of lysosomes in cellular clearance pathways.

It has recently been discovered that lysosomal function is regulated by a gene network named Coordinated Lysosomal Expression and Regulation (CLEAR), mainly activated by a master transcription factor, transcription factor EB (TFEB). TFEB is part of a protein complex named LYsosome NUtrient Sensing (LYNUS), which also comprises mammalian target of rapamycin complex 1 (mTORC1), and this machinery is attached to lysosomal membrane. This kinase complex is able to sense lysosomal nutrient content and send signals to the nucleus; in nutrient-rich conditions, mTORC1 phosphorylates TFEB and it becomes inactive in cytoplasm. Due to adverse conditions such as starvation or exercise, TFEB is dephosphorylated by calcineurin, enabling it to translocate to the nucleus and regulate expression of various genes related to autophagy, fatty acid beta-oxidation, lipophagy, and lysosomal biogenesis pathways (10–12).

Transcription factor EB activation seems to be a response to lysosomal stress; this lysosome-to-nucleus signaling allows lysosomes to adapt to different physiological and pathological conditions (10, 13). As an example of these adverse conditions in LSD, deficiency of α-l-iduronidase in mucopolysaccharidosis type I destabilizes lysosomal Ca^2+^ and H^+^ homeostasis, leading to lysosomal membrane permeabilization (14, 15), which triggers different pathogenic cascades. In addition, autophagy blockage has also been described in many LSD, since autophagosome–lysosome fusion is impaired due to lysosomal dysfunction (1). This context of lysosomal alterations in LSD may be regarded as chronic lysosomal stress, which is likely to be associated with changes in TFEB signaling and regulation of CLEAR network genes.

Direct binding of TFEB to a 10-base site (TCACGTGA) found in promoters of several lysosomal genes is required for the expression of CLEAR genes. TFEB expression has already been shown to be downregulated by a miRNA (*miR-128*) (10), emphasizing the importance of these molecules for the regulation of CLEAR genes expression and, consequently, lysosomal function. Furthermore, expression of each CLEAR gene may be regulated by different miRNA [and/or by other non-coding RNA (ncRNA)], bringing additional layers of complexity to this system.

Therefore, as lysosomal function depends on this gene network, it seems very likely that ncRNA play an important role in fine tuning of lysosomal genes expression, modulating disease onset, severity, and progression. In the next session, we will discuss current data on the literature regarding the association of ncRNA and LSD.

## LSD and ncRNA Studies

The paradigm DNA–RNA–protein has been modified long time ago, since the discovery of RNA molecules capable of acting at transcription, translation, and RNA modification processes [reviewed by Cech and Steitz (16)]. These RNA molecules, the so-called ncRNA, are a functional class of RNA involved in the regulation of several cellular processes, such as repression or enhancement of gene expression, protection against deleterious nucleic acids, and histone and DNA modifications [reviewed by Yang et al. (17)]. They have also been implicated in a number of human diseases as cancer, neurodegenerative and autoimmune disorders, modulating disease onset, progression, and prognosis. The most known classes of regulatory ncRNA are miRNA, siRNA, piRNA, and lncRNA, each having their own characteristics and functions [reviewed by Zhou et al. (18)].

To date, all studies regarding the association of ncRNA and LSD available in the literature have focused only on miRNA, which are small ncRNA (19–24 nucleotides transcripts) that direct posttranscriptional silencing of complementary mRNA targets, through assembling with Argonaute proteins in the miRNA-induced silencing complexes (miRISC). This mechanism regulates gene expression by repressing translation or destabilizing the mRNA molecule. It has been demonstrated that one miRNA is able to regulate the expression of hundreds of genes, making these molecules crucial for almost all biological processes (19, 20).

The first report of a connection between LSD and ncRNA was published by Ozsait et al. (21), in which they performed a miRNA array in fibroblasts of patients with Niemann–Pick type C (NPC), a lipid storage disease caused by mutations in *NPC1* or *NPC2* genes. The miRNA profile revealed that among 365 evaluated miRNA, 3 of them were upregulated and 38 were downregulated in NPC cells. The differentially expressed miRNA are predicted to control the expression of genes involved with metabolism of cholesterol and other lipids, also involved with endocytosis, cytoskeleton organization, and ubiquitin pathways. Interestingly, only few miRNA (*miR-26b, miR-19a*, and *miR-19b*) target genes which are directly related to metabolism of cholesterol, considering that the prior cause of NPC disease is the cholesterol storage. A similar study was performed by Arora et al. (22), in which a screening of siRNA was carried out in order to identify modulators of cholesterol accumulation in two NPC cell lines. Cells were transfected with selected siRNA individually and were stained with filipin, a fluorescent marker of cholesterol accumulation. Changes in filipin staining were found in NPC fibroblasts transfected with siRNA that targeted low-density lipoprotein receptors (LDLR, APOL1, NPC1, NPC1L1, NPC2, STARD3, STARD4, and RAB9A) and sphingomyelin phosphodiesterase 1, evidencing the role of these genes in lipid metabolism.

Gaucher disease, a relatively frequent LSD caused by deficiency of lysosomal glucocerebrosidase (Gba), a *GBA1* encoded enzyme that degrades glucocerebroside into glucose and ceramide (23), has already been evaluated under the ncRNA perspective. Siebert et al. (24) performed a screening of miRNA involved in the modulation of Gba in fibroblasts of Gaucher patients and showed that enzyme activity was specially upregulated by five and downregulated by three miRNA. Then, cells were transfected with these selected miRNA in order to evaluate gene transcription and protein expression when compared to negative control transfected cells. Two miRNA (*miR-195-5p* and *miR-16-5p*) strongly upregulated *GBA1* expression, while three miRNA (*miR-127-5p, miR-19a-5p*, and *miR-1262*) downregulated *SCARB2*, an important membrane receptor involved in Gba availability. One of them, *miR-127-5p*, downregulated Gba activity and also its levels, since it was also able to downregulate *SCARB2* expression.

On the other hand, Ginns et al. (25) studied a murine model of Gaucher disease, treated with a Gba inhibitor (conduritol β-epoxide). It is known that patients with Gaucher disease present a significant risk factor for the development of Parkinson disease, which is reinforced by previous data showing α-synuclein accumulation and signs of neuroinflammation in Gaucher animal models. Authors have detected changes in the expression of miRNA in ventral mesencephalon of the animal model: two miRNA were upregulated (*miR-29b* and *miR-142*) while *miR-let7b* was downregulated, all involved with the modulation of genes related to inflammatory response. The study suggested that Gba deficiency may influence signaling cascades involving neuroglia, α-synuclein, and miRNA, all contributing to early pathogenesis, synaptic dysfunction, and neurotoxicity, common both to Gaucher and Parkinson diseases.

Frankel et al. (26) demonstrated that *miR-95* controls the expression of sulfatase-modifying factor 1 (SUMF1) gene, which encodes an activator of sulfatases. These enzymes are essential for the catabolism of glycosaminoglycans and are deficient in multiple sulfatase deficiency (MSD), as a consequence of mutations in *SUMF1* gene. They have observed an increase in *SUMF1* expression and in its enzymatic activity as a result of *miR-95* inhibition. In addition, *miR-95* transfected cells showed higher GFP-LC3II positive cells and the accumulation of early endosomes, multivesicular bodies, autophagic substrates, and autophagic vacuoles, which reinforces the interference caused by *miR-95* in endocytic and autophagic mechanisms. These data were confirmed in cells of MSD patients, since authors proved that inhibition of *miR-95* could raise residual *SUMF* expression and also SUMF1 activity.

Taken together, these data emphasize the role of miRNA in modulating specific pathogenic cascades involved in the physiopathology of different LSD. As discussed in the first session, there is still a lot to learn about specific secondary alterations due to enzyme deficiency in this group of diseases, and the recent discovery of CLEAR network shows us additional layers of potential regulators and/or modulators of gene expression in a number of processes associated with lysosomal function, which reinforces the importance of studying miRNA and other ncRNA in this context. The next session will describe available tools for analyzing ncRNA, which could be used for studying these molecules in LSD models or patients.

## Tools for ncRNA Evaluation

The discovery of ncRNA and the evaluation of their functions have improved significantly in the last 15 years due to the raise of new questions and the advance of molecular biology technologies (27, 28). ncRNA are already associated with important functions in genetic and epigenetic regulation, such as modulation of chromatin modification, DNA methylation, transcription, and translation processes. Therefore, specific techniques must be used in order to assess each one of the possible levels of regulation. The most common approach is to perform high throughput analysis in order to identify alterations in ncRNA profile and then investigate those of interest (29, 30). However, bioinformatics is extremely implicated in ncRNA studies since it is relevant in identification of new ncRNA sequences and in predicting their targets by *in silico* approaches [reviewed by Krzyzanowski et al. (31)].

The first step in ncRNA assessment is generally based on some previous data, e.g., an unknown underexpression of a gene or an unexpected methylation of a gene promoter, or an interest in evaluating their general profile under experimental conditions. Thus, the most common approaches to evaluate ncRNA status are transcriptome analysis, PCR arrays, microarrays, and other screening assays (29, 30). After gathering these data, bioinformatics analysis must be carefully performed in order to obtain reliable results, since it can highlight some ncRNA molecules and also predict their interaction with other molecules, such as miRNA–mRNA base-pairing and/or lncRNA contact with histones, which may turn plausible the explanation of some specific event (31, 32).

Once verified the possible relevance of ncRNA in a context, it becomes necessary to perform validation experiments. Generally, the most common validation method is quantitative RT-PCR because of its power to easily quantify RNA species from several samples at the same time. Alternatively, Northern blot can be performed in order to evaluate ncRNA profile in different conditions (29). There are also specific techniques to evaluate the role of ncRNA in mRNA regulation, such as luciferase activity assay for miRNA and piRNA and the utilization of miRNA mimic, inhibitor, and/or sponge in cell culture (33, 34). On the other hand, it may be interesting to assess ncRNA importance in protein expression and in chromatin modifications by Western blot or chromatography approaches and RNA immunoprecipitation (RIP), RIP-Chip, and ChIRP, respectively (29, 33).

## Closing Remarks and Future Perspectives

The study of LSD and ncRNA association is just beginning. Until now, the primary focus of the authors was the evaluation of miRNA profile in the context of LSD, since there are reports showing that alterations of miRNA expression in several diseases may be involved in the modulation of their outcome, as well as being used as a disease biomarker (35–38). Secondarily, the utilization of specific siRNA was a pronounced strategy to evaluate the impact of several genes in cholesterol metabolism, elucidating their role in NPC pathogenesis. Nevertheless, there are no articles comprising the role of lncRNA, piRNA, and other important members of ncRNA in LSD.

Considering the plethora of secondary pathways involved in LSD, including CLEAR network dysregulation and neurodegeneration pathways, it seems very likely that ncRNA might play an important role in the regulation of these mechanisms and eventually in disease physiopathology, contributing to phenotypic variability. Transcriptome analysis has the potential to reveal RNA molecules affected by LSD, which may enlighten some poorly understood mechanisms and enable the comparison of mRNA and ncRNA profile in different LSD. Therefore, efforts in performing ncRNA analysis on well-established models of LSD should be taken in order to better comprehend their complexity and possibly discover potential biomarkers and therapeutic targets.

## Author Contributions

All authors listed contributed equally to the work and approved it for publication.

## Conflict of Interest Statement

The authors declare that the research was conducted in the absence of any commercial or financial relationships that could be construed as a potential conflict of interest.
